# Operation of Hybrid Membranes for the Removal of Pharmaceuticals and Pollutants from Water and Wastewater

**DOI:** 10.3390/membranes12050502

**Published:** 2022-05-08

**Authors:** Mónica Vergara-Araya, Henning Oeltze, Jenny Radeva, Anke Gundula Roth, Christian Göbbert, Robert Niestroj-Pahl, Lars Dähne, Jürgen Wiese

**Affiliations:** 1 Department for Water, Environment, Construction, and Safety, Magdeburg-Stendal University of Applied Sciences, Breitscheidstr. 2, 39114 Magdeburg, Germany; henning.oeltze@h2.de (H.O.); juergen.wiese@h2.de (J.W.); 2 Nanostone Water GmbH, Am Bahndamm 12, 38820 Halberstadt, Germany; jenny.radeva@nanostone.com (J.R.); anke-gundula.roth@nanostone.com (A.G.R.); christian.goebbert@nanostone.com (C.G.); 3 Surflay Nanotec GmbH, Max-Planck-Str. 3, 12489 Berlin, Germany; r.niestroj-pahl@surflay.com (R.N.-P.); l.daehne@surflay.com (L.D.)

**Keywords:** ceramic membrane, hybrid membrane, LbL coating, removal of pharmaceuticals, removal of pollutants, membrane cleaning

## Abstract

Hybrid ceramic membranes (i.e., membranes with a layer-by-layer (LbL) coating) are an emerging technology to remove diverse kinds of micropollutants from water. Hybrid ceramic membranes were tested under laboratory conditions as single-channel (filter area = 0.00754 m^2^) and multi-channel (0.35 m^2^) variants for the removal of pharmaceuticals (sulfamethoxazole, diclofenac, clofibric acid, and ibuprofen) and typical wastewater pollutants (i.e., COD, TOC, PO_4_-P, and TN) from drinking water and treated wastewater. The tests were conducted with two low transmembrane pressures (TMP) of 2 and 4 bar and constant temperatures and flow velocities, which showed rejections above 80% for all the tested pharmaceuticals as well for organic pollutants and phosphorous in the treated wastewater. Tests regarding sufficient cleaning regimes also showed that the LbL coating is stable and resistant to pHs between 2 and 10 with the use of typical cleaning agents (citric acid and NaOH) but not to higher pHs, a commercially available enzymatic solution, or backwashing. The hybrid membranes can contribute to the advanced treatment of water and wastewater with low operational costs, and their application at a larger scale is viable. However, the cleaning of the membranes must be further investigated to assure the stability and durability of the LbL coating.

## 1. Introduction

The presence of pharmaceuticals in natural waters is a problem of increasing concern. Pharmaceuticals, which are by nature biologically active and hydrophilic, are not usually completely mineralized in the wastewater treatment process [1]. As a result, pharmaceuticals can accumulate in water bodies, leading to negative consequences for ecosystems and humans [2].

To protect the environment and people’s health, increasing attention is being paid to regulations for micropollutant removals and the advanced treatment of wastewater, i.e., treatment beyond organic and nutrient removals. Wastewater treatment plants (WWTP) are particularly designed to remove organic matter and nutrients from wastewater to prevent the eutrophication of water bodies and protect human health and the environment. In the last decades, a necessity to improve the quality of natural water bodies has led to the sharpening of wastewater discharge norms in different regions in the world, putting a special emphasis on the removal of nutrients, such as nitrogen and phosphorous, with strict discharge norms of ≤2 mg/L for ammonium nitrogen (NH_4_-N) and total phosphorous (TP) [3,4]. The removal of nutrients from wastewater is usually carried out biologically and/or chemically; therefore, these discharge values can be challenging even for experienced operators in highly technical WWTPs. The application of membranes for this purpose is usually limited due to a high power consumption and large water volumes as nanofiltration (NF) technologies or reverse osmosis (RO) are required due to the small molecular weight of nutrient molecules (i.e., NH_4_^+^ = 18 g/mol; NO_3_^−^ = 62 g/mol; and PO_4_^3−^ = 94.97 g/mol).

The use of membrane technologies for the treatment of water and wastewater has grown considerably in the last decades as the pressure on freshwater resources has increased and the technologies have become smaller, more efficient, and more economically viable [5].

Moreover, the use of membranes for the advanced treatment of water and wastewater has become increasingly relevant due to its main advantage: the absence of residual products in water after such treatments in comparison to other alternatives, such as ozone, UV light, and activated carbon. The investment prices for membranes, which were prohibitive in the past, have evolved in the last years, making membranes a more competitive technology.

In the treatment of wastewater, membrane filtration systems, such as reverse osmosis (RO) and forward osmosis (FO), have shown high rejections of the typical pharmaceuticals found in WWTPs [6]. Other combinations of membrane systems, such as microfiltration (MF) and RO, have been used to remove pesticides and pharmaceuticals from wastewater for water reclamation purposes [7]. NF and RO systems require high operational pressures (>5 bar), therefore representing high operational costs.

Hybrid treatment systems, such as membrane biological reactors (MBRs), are widespread technologies; however, there is no concluding evidence indicating that MBRs are better at removing pharmaceuticals than conventional biological treatment systems [6].

Ceramic membranes show advantages in comparison with polymeric membranes in terms of a high-temperature stability, a fouling resistance, and low maintenance requirements [8]. Furthermore, ceramic membranes, which were limited in their pore sizes in the past, can be coated with polyelectrolyte layers to go from characteristics matching ultrafiltration closer to nanofiltration, improving the membranes’ selectivity [9]. Previous studies of the membranes described herein showed a molecular weight cut-off (MWCO) of approximately 275 g/mol [10]. Moreover, the development of the technology has made ceramic membranes’ application possible even at low operational pressures.

As an alternative to nanofiltration membranes, based on mainly functionalized polyamides, polymeric [11] or ceramic membranes [12] can be coated with nanometer-thin polyelectrolyte layers using the layer-by-layer (LbL) approach [13]. In previous studies, polyelectrolytes, such as cationic poly(diallyldimethylammonium chloride) (PDADMAC) and poly(allylamine hydrochloride) (PAH), and the anionic salt of poly(styrenesulfonate) (PSS) were applied. This means that not only were the pore sizes reduced but an additional selectivity was provided due to the type of polymer, resulting in a different surface functionality, such as hydrophobicity, hydrogen bonding, charge polarity, and a density of the membrane surface. The LbL technology in membrane filtration has been tested successfully under different conditions for salt retention, the removal of antibiotic resistance genes [14], and the removal of pharmaceuticals from aqueous solutions [10], among other functionalities.

Compared to the well-established nanofiltration (NF) membranes, LbL-coated membranes show several potential advantages. LbL-coated polymeric membranes for commercial applications were first developed by Surflay Nanotec GmbH and Pentair X-Flow in the EU project LbLBRANE [15]. Due to an alternating deposition of polycation and polyanion with thicknesses of 1-4 nm from aqueous solutions, LbL-coated membranes’ properties can be modified easily in a very controlled, simple, and reproducible way. There are more than 50 different polyanions and polycations available on the market, with different materials, molecular weights (MWs), charge densities, functionalities, pK values, hydrophobicities, and persistence lengths. Furthermore, film thicknesses can be controlled by conditions of assembling, such as ion strength, pH, and layer number. In such multi-layer assemblies, each layer can be formed by using a different polyelectrolyte, creating a broad combination of different functionalities [16].

Due to the use of purified polyelectrolytes, there are no problems with dangerous monomers possibly remaining in the filtrated aqueous solutions.

LbL-coated membranes have shown that their permeability (up to 15 L/m^2^ h bar) is slightly higher and their necessary TMP is remarkably less than for nanofiltration membranes [17].

In the operation of hybrid ceramic membranes, several aspects play a role. Transmembrane pressure (TMP) must be kept as low as possible to reduce operational costs but shows an economically viable throughput. Operational pressures between 2 and 10 bar have been tested in the past with successful results. Operations in cross-flows have been shown to make the most of the LbL coating, while the dead-end operational principle appears to not be compatible with the rejection of substances below the degree of UF [9].

The temperature of the liquid feed as well as the flow velocity also play roles in the membranes’ permeabilities and performance in general.

Another central aspect is the cleaning regime of hybrid membranes. During the long-time use of membranes, fouling can appear, affecting a membrane’s permeability and operational costs negatively. Therefore cleaning in place (CIP) is a routine process in membrane operations. There are two main forms of CIP: mechanical and chemical. Mechanical cleaning is used for reversible fouling and chemical cleaning is used for irreversible fouling [18]. Ceramic membranes tend to be less prone to fouling and are perfectly compatible with typical cleaning methods (i.e., cleaning with acidic or caustic chemicals, enzymatic solutions, and backwashing). However, in the case of hybrid membranes with polyelectrolyte coatings, traditional cleaning methods can eventually affect the LbL coating and the membranes’ performance and must be further evaluated. It was tested in the past, and it was found that solutions containing NaOCl can damage the coating containing PAH as it oxidizes exposed primary amines, leading to a change in the coating’s characteristics [19].

Up to now, only LbL-coated polymeric PES membranes are available on the market. Ceramic membranes have the big advantage of remarkably longer life-times. However, their pore size distributions are larger than for polymeric membranes, thus making a controlled covering of the pores with LbL films more difficult [12]. In contrast to membranes with polymeric support, long-term use is also possible for the LbL-coated ceramic membranes as the coating can be removed completely and changed without damaging the membrane [12]. Therefore, the further development of LbL-coated ceramic membranes as nanofiltration membranes is addressed and presented in this paper.

## 2. Materials and Methods

### 2.1. Layer-by-Layer Technology

Poly(allylamine hydrochloride) (PAH; MW of 150.000; 40 wt %) was received from Nittobo Medical Co., Tokyo, Japan. Sodium poly(styrenesulfonate) (PSS; MW of 1.000.000) was received from Merck Sigma Aldrich, Germany. Magnesium sulfate and sodium chloride were purchased from Carl Roth, Karlsruhe, Germany. Diclofenac sodium salt was received from TCI Chemicals, Eschborn, Germany.

**Figure 1:**
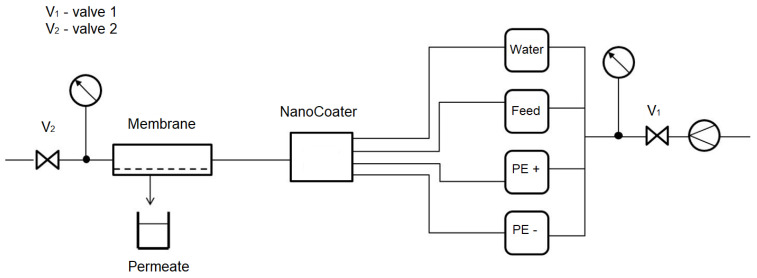
Principle of the NanoCoater process to generate hybrid membranes using the LbL technology [14].

For polyelectrolyte coatings, a device named NanoCoater developed by Surflay (see Figure 1) was used. Electronically, timer-controlled valves in the device guide coating solutions to a membrane. The coating program contains coating and washing steps and can automatically run various cycles to apply multiple layers to the membrane.

In detail, the first polyelectrolyte that was applied to the membrane was the polycation PAH. Without any coating, the ceramic membrane had a negative charge. The coating solution consisted of 1 g/L of a polyelectrolyte with 0.5 M of NaCl and a defined pH value fixed by using 50 mM of a buffer solution. The contact time was 5 min. Next, the membrane was washed 2 × 5 min with ultrapure water. The following step was coating with the polyanion PSS. Similar to the polycation, the coating solution consisted of 1 g/L of a polyelectrolyte with 0.5 M of NaCl of same pH. Contact time was 5 min. Again, the membrane was washed twice with ultrapure water. Then, the coating loop was repeated 8 times to apply 8 double layers of polyelectrolyte onto the membrane.

After the coating procedure, the membrane was submerged in ultrapure water overnight to remove the excess coating solution and excess NaCl. Subsequently, the membrane was ready to be analyzed. The membranes being used in this comparison were coated with (PAH/PSS)_8_.

### 2.2. Membrane Descriptions

**Figure 2:**
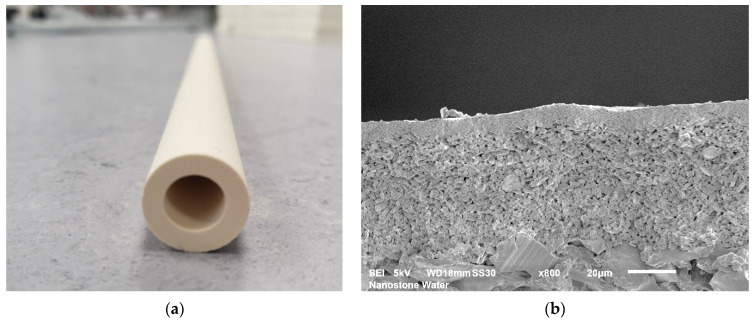
Single-channel ceramic segment: (**a**) monotube ceramic membrane with dimensions of length of 300 mm; height of 14.0 mm; wall thickness of 3.0 mm; and channel diameter of 8.0 mm and (**b**) SEM picture of the ceramic membrane. Shown is the substrate being coated with two defined ceramic membranes to reach a final pore size of 30 nm.

The hybrid membrane consisted of a ceramic part and a polymeric part. A single-channel alumina ultrafiltration ceramic membrane was used as the substrate for the polyelectrolyte coat. A defined ceramic filtration membrane bearing a pore size of 30 nm was applied on the inner side of the channel (see Figure 2) and separated small particles, viruses, bacteria, and colloids. At the same time, the ceramic membrane layer carried the applied layer-by-layer polyelectrolyte coat, and its properties played a key role in the compatibility and robustness of the hybrid membrane. The properties of the ceramic ultrafiltration membrane were previously investigated and published in [10]. The measured pore size of the uncoated ceramic membrane was between 59 and 215 nm, and after LbL coating, the estimated pore size was between 1.5 and 4.4. nm. The molecular weight cut-off for the hybrid membranes was 270 g/mol. A zeta potential pH scan confirmed that the application of polyelectrolytes on the surfaces of the ceramic membranes led to a switching of the zeta-potential trend and its isoelectric point [10].

### 2.3. Substance Analysis

#### 2.3.1. Chromatographic Analysis of Pharmaceuticals

An analysis of micropollutants via HPLC with a diode array detector (DAD) was carried out at the Magdeburg-Stendal University of Applied Sciences. The analysed substances were sulfamethoxazole, diclofenac, clofibric acid, and ibuprofen. Their main characteristics are described in Table 1. The detection of the four trace substances was integrated into one chromatographic method based on Macherey–Nagel application no. 11777 [20].

The DAD detector that was used was operated at a wavelength of 200 nm, and a limit of detection (LOD) of 5 µg/L per trace substance was achieved. Due to this relatively high LOD and to measure the membrane permeate, the micropollutant concentrations on the feed samples were augmented by 100 µg/L for each substance. This was carried out in all the experiments with drinking water and most of the experiments with treated wastewater.

The measurement uncertainties for values above 50 µg/L (i.e., feed) were around 8% on average. For the measurements below 25 µg/L (i.e., permeate), the measurement uncertainty increased to 27%.

#### 2.3.2. Mass Spectrometry for the Analysis of Pharmaceuticals

Some experiments with treated wastewater were carried out without pharmaceutical augmentations. To be able to measure values below the LOD of the chromatographic method, as described above, the samples were sent to an external laboratory to be analyzed by using mass spectrometry (MS) as analogy of method DIN 38407-47: 2017-07 (DIN “Methods for the determination of selected active pharmaceuticals and other organic substances in water and wastewater. Method using high-performance liquid chromatography and mass spectrometric detection (HPLC-MS/MS or -HRMS) after direct injection”).

#### 2.3.3. Analyses of Wastewater Parameters

The main pollutants in wastewater are organic matter and nutrients. Wastewater samples were analyzed with LCK cuvette tests from Hach Lange. The sum of organic substances was measured as COD with the test LCK-1414 (LOD = 5–60 mg O_2_/L), TOC with Dimatoc 2000, and thermal-catalytic oxidation with NDIR detection. Nitrogen was measured as total nitrogen (TN) with the same method and device as TOC. Phosphorous was measured as total phosphorous (TP) with the LCK 349 tests (LOD = 0.05–1.5 mg P/L).

### 2.4. Rejection Tests with Hybrid Membranes

Rejection tests were carried out for single and multi-channel membranes. The hybrid single-channel membranes (filter area = 0.00754 m^2^) were tested in a laboratory filtration skid (Filtration Plant 1), which was designed to examine single-channel membranes according to filtration application standards. The plant had a feed container of 5 L of the solution, a pump to feed the membrane, and a needle valve to control the operational pressure. The membranes were operated using a cross-flow process (crossflow velocity = 2.15 m/s) and a heat exchanger to maintain a constant solution temperature.

**Figure 3:**
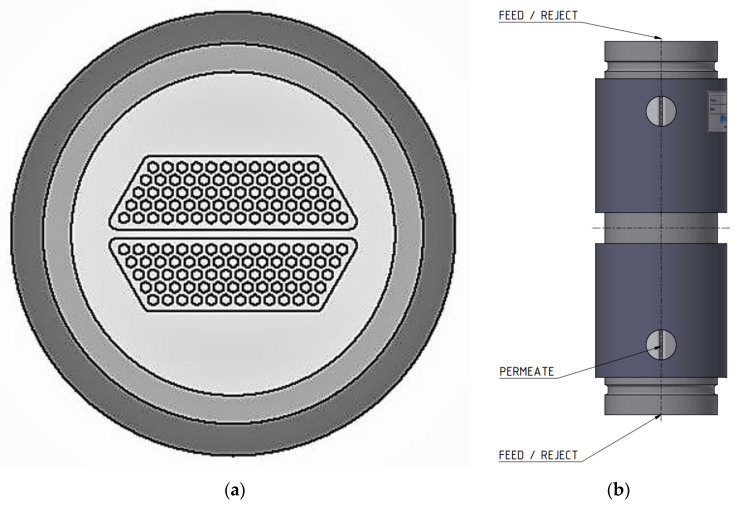
(**a**) Multi-channel ceramic membrane module cross-section and (**b**) multi-channel ceramic membrane module housing, indicating feed, reject, and permeate ports.

In scale-up experiments, larger ceramic membrane modules of 0.35 m^2^ surface areas (see Figure 3a) were tested in a larger laboratory filtration plant (Amafilter, Lochem, The Netherlands), also called Filtration Plant 2, which is operated under the cross-flow principle. Just as the laboratory filtration skid, this plant was equipped with a heat exchanger to maintain a constant feed temperature. In all experiments, the inflow rates of the membrane modules for both systems were approximately 400 L/h each.

The characteristics of the membranes that were used are detailed in Table 2.

The tested membranes (both single-channel and multi-channel) were coated with eight layers of PAH/PSS ((PAH/PSS)_8_) at a constant solution temperature of 15 °C, constant overflow velocity as described in Table 2, and two different transmembrane pressures (TMPs) of 2 and 4 bar. The experiments were carried out with two different matrices: drinking water (pH = 7.56; electrical conductivity = 587 µS/cm; and total hardness = 2.1 mmol/L CaCO_3_ at the production site [26]) and effluent from a wastewater treatment plant (WWTP; class size 5 with >100,000 population equivalents).

A summary of operational parameters of the different tests is in Table 3. The values shown represent the averages of between 2 and 6 individual experiments (n = 2–6).

### 2.5. Membrane Cleaning

A routine aspect of membrane operation is cleaning in place (CIP) to restore the membrane’s performance after fouling. The two main forms of CIP, mechanical and chemical, were tested. The chemical cleaning was carried out with acidic, caustic, and enzymatic cleaning solutions under the conditions described in Table 4.

The objectives of these tests were to test the stability and durability of the membrane coating, i.e., to observe, and to test whether the membrane coating’s performance was affected. It is important to mention that the experiments were not designed to remove a particular type of fouling because fouling was not observed during the experimental period. The evaluations of the membrane’s performance were carried out by comparing rejections of pharmaceuticals and permeabilities before and after the membrane’s cleaning.

#### 2.5.1. Chemical Cleaning

For the chemical cleaning of the membrane, different methods were tested and are described in Table 4. The tests were carried out in drinking water with a pharmaceutical spiking as described in Section 2.3.1 and a TMP of 4 bar. Three main cleaning approaches were tested: cleaning with acidic, cleaning with caustic, and cleaning with enzymatic solutions. The chemical dosings during the utilization of citric acid and NaOH were intended to obtain a target pH, as shown in Table 4. In the case of the enzymatic cleaner, P3-Ultrasil 53, an enzymatic cleaner containing mainly EDTA and phosphates, was used. The target concentrations were based on the parameters noted by several authors [27,28,29]. The performed cleaning was static, conducted by submerging the membrane into the cleaning solution for 30 min; afterwards, it was washed with distilled water for 10 min at 4 bar and constant overflow velocity as described in Table 2.

The stability of the LbL coating was determined by comparing rejections of different pharmaceuticals before and after the cleaning process with the same sample liquid. The filtration process was carried out with two filtration steps, a constant overflow velocity, a TMP of 4 bar, and a measurement interval of 15 min.

#### 2.5.2. Backwash

A backwash is a standard process of mechanical cleaning with which the flow direction is reversed in reference to the usual flow direction. The objective is to loosen and remove impurities and so-called filter cakes. A backwash is often carried out in situ and sometimes only as a backwash pulse, a very short backwash under high pressure. The advantage is that the system does not have to be interrupted and only a little time has to be spent on the cleaning process.

Rejection tests with single-channel hybrid membranes in the Filtration Plant 1 were carried out before and after membrane backwashing to observe changes in the membrane’s performance. The backwash experiments were performed in a sophisticated backwash skid, which was designed and built at the Magdeburg-Stendal University of Applied Sciences and is based on tubes made of PE-RT (polyethylene reinforced with aluminum (coated inside and outside with PE-RT)) and corrosion-resistant brass, which makes it ideal for tests with chemicals.

The backwash process was carried out by feeding distilled water from the outer surface of the membrane to the inside, the coated layer. The backwash duration was kept the same for all three experiments; only the backwash pressure was increased. After the backwash, the membrane was reinstalled in Filtration Skid 1 and two filtration steps were carried out at constant overflow velocities as described in Table 2, TMPs of 2 bar and intervals of 15 min [8] to compare results and verify the integrity of the LbL coating.

## 3. Results and Discussion

### 3.1. Rejection Experiments of Pharmaceuticals

**Figure 4:**
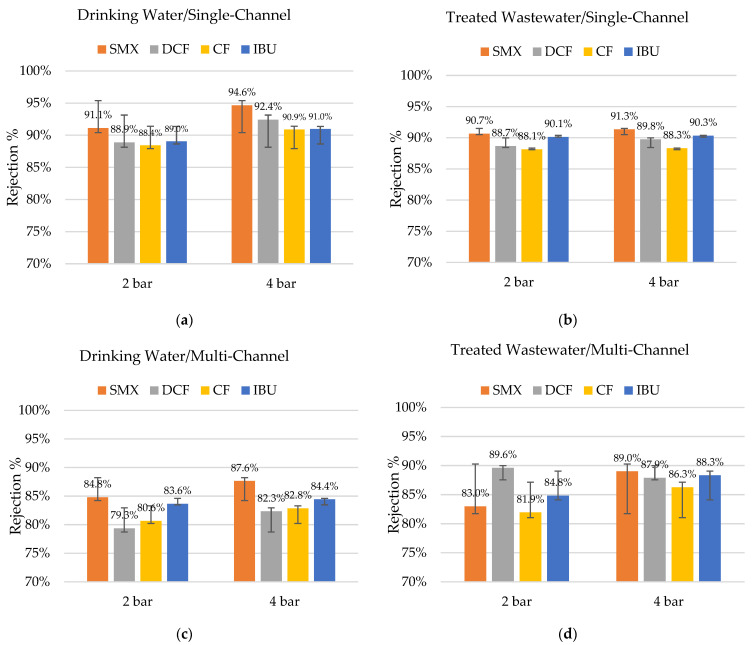
Rejection percentages of sulfamethoxazole (SMX), diclofenac (DCF), clofibric acid (CF), and ibuprofen (IBU), with pharmaceuticals spiking under different TMPs (**a**) drinking water with single-channel hybrid membrane (n = 3); (**b**) treated wastewater with single-channel hybrid membrane (n = 5–6); (**c**) drinking water with multi-channel hybrid membrane (n = 3); and (**d**) treated wastewater with multi-channel hybrid membrane (n = 2).

The rejection of pharmaceuticals was very high (i.e., >79%) in all the studied cases, as shown in Figure 4. The trend shows that in experiments with drinking water as a matrix, the rejections were slightly higher (up to 3.3%) compared to using treated wastewater, but the difference was not significant. This means that the many other compounds present in the treated wastewater did not interfere with the rejection of the studied pharmaceuticals.

It is possible to observe that the average rejections obtained in the single-channel hybrid membranes were slightly higher when compared to the multi-channel equivalents. Despite the smaller diameter of the individual membrane channel, the overall cross-sectional area of the multi-channel membrane was higher (Table 2). Therefore, the cross-flow velocity for the multi-channel membrane was lower, which possibly contributed to a lower rejection of pharmaceuticals [30]. The inflow rates of the membrane modules for both systems were approximately the same (400 L/h). The difference may have also been related to the differences in the coating processes (upscaling) and the utilized pilot plant. The coating process is not standardized for multi-channel membranes yet; therefore, each membrane must be seen as a unique test specimen—a prototype.

Until today, the coating process of single-channel membranes has been well defined and a standardized protocol. In comparison to a multi-channel membranes, the process has been much simpler and less susceptible to coating flaws due to higher channel diameters. Multi-channel membranes, described here, were coated for the first time to be used in this set of experiments. The continuous development of the coating technology and the experience gained in the coating process may be subject to alterations and changes in the future.

Moreover, the utilization of multi-channel membranes in an adapted second pilot skid (Filtration Plant 2) for multi-channel membranes was commissioned and set up. Therefore, the experience gained in the operation of the multi-channel test skid was limited in comparison with the experience gained during the execution of experiments with the first pilot plant to test single-channel membranes.

Despite these challenges and potential sources of error, the rejection rates for the multi-channel membranes are high but can be still optimized.

Surprisingly, the rejection rates for diclofenac were slightly lower despite it being the largest molecule of the selected four with an MW almost 10% above the expected MWCO_90_ of the studied membranes. Moreover, the rejection rates for ibuprofen were higher than for the clofibric acid, even considering that ibuprofen is the smallest molecule (ca. 24% below the expected MWCO_90_). This is a clear indicator that the size of the molecule is only one factor determining its rejection. The formal charges of all the tested pharmaceuticals are zero, but their electrochemical characteristics differ. Sulfamethoxazole, for example, has a significantly larger polar surface than the other three studied substances and was the molecule with the highest rejection rates under almost all the tested conditions.

The rejection mechanisms in hybrid (ceramic) membranes are complex and still not fully understood, but the results presented here can possibly contribute to a better understanding of the involved phenomena and mechanisms.

A slightly higher retention with a 4 bar TMP was observable. According to the literature [31], there is an increase in rejection with an increase in permeate flux, as observed in this set of experiments. Due to the linear correlation of permeate flux and pressure, the salt concentration in the permeate was diluted with increased pressure as the rejection was not linear to the pressure. Therefore, the rejection increased with elevated pressure.

**Figure 5:**
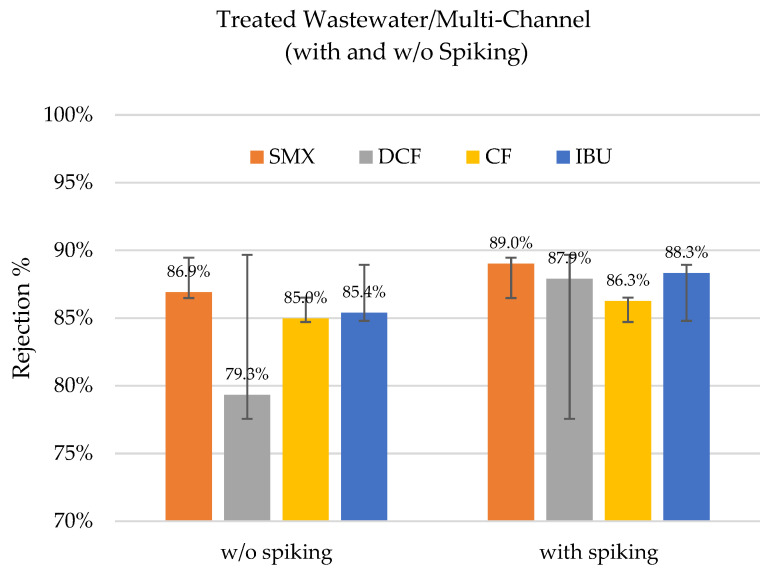
Rejection percentages of sulfamethoxazole (SMX), diclofenac (DCF), clofibric acid (CF), and ibuprofen (IBU) with spiking and without spiking in the treated wastewater with a multi-channel hybrid membrane at a TMP of 4 bar (n = 2).

Experiments with and without the spiking of the four pharmaceuticals were carried out to test if the concentrations of the substances influenced the rejection rates (see Figure 5). It is important to remember that the values obtained in the experiments with spiking were obtained via HPLC and the results without spiking were obtained with MS, as described in Section 2.3.1 and Section 2.3.2.

All the observed rejection rates were above 79%. In the event of spiking, the rejection rates even improved slightly for sulfamethoxazole, clofibric acid, and ibuprofen. Meanwhile, there was a larger difference for diclofenac. This indicates that the initial concentration in the feed, i.e., the concentration gradient, could play a role in the rejection rates, but its influence was not significant in the overall process. The concentrations in the feed and permeate are presented in Table 5.

The method applied at the external laboratory, which analysed the samples without spiking (DIN 38407-47: 2017-07), was recently replaced by the latest DIN norm (DIN EN ISO 21676:2022-01).

### 3.2. Rejection Tests of Common Wastewater Pollutants

**Figure 6:**
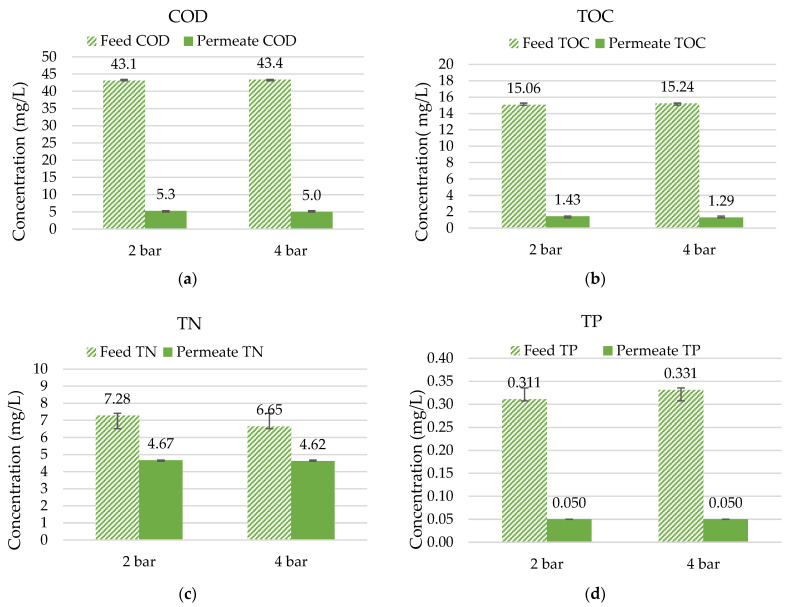
Rejection percentages in multi-channel hybrid membranes with treated wastewater (n = 4) of (**a**) COD; (**b**) TOC; (**c**) TN; and (**d**) TP.

The rejection of organic compounds, measured as the sum parameters COD and TOC (see Figure 6), was very high, reaching about 77% for COD and more than 90% for TOC. Organic molecules present in wastewater vary in size, but they are present as particulate and dissolved substances. It was expected that all particulate CODs would be retained in the filtration, while some of the dissolved COD would be detectable in the permeate. Moreover, organic pollutants tend to be larger than the nutrients present in wastewater and usually have a positive load, contributing to the rejection mechanism provided by the polyelectrolyte coating.

The removal of nutrients was also observed in the experiment. A nitrogen removal of between 30 and 36% could be confirmed in the experiments carried out. In general, more than 75% of the total nitrogen present in the effluent of the WWTP was in the form of nitrate (MW = 62 g/mol), a molecule that is well below the expected MWCO_90_ of the hybrid membrane.

Phosphorous measurements were challenging due to their very low concentrations in the feed. WWTPs in Germany must remove phosphorous to reach values below 1 mg/L in the effluent depending on the plants’ sizes and regions. Therefore, cuvette tests often reach their LODs in permeate solutions. Consequently, when the permeate solution shows values below the LOD for total phosphorous (TP; 0.05 mg/L), the lowest value of the measurement range is listed.

The results show that an overall improvement in the quality of treated wastewater can be obtained with the application of hybrid ceramic membranes. This can help to meet the standard at very strict discharge limits and to also open the possibility of reusing the treated wastewater. The reuse of treated wastewater is not widespread in Germany but it is developing quickly due to the challenges imposed by climate change and water scarcity in some designated regions. As the reuse of treated wastewater becomes increasingly important in different regions worldwide, this technology is promising to serve this purpose.

**Figure 7:**
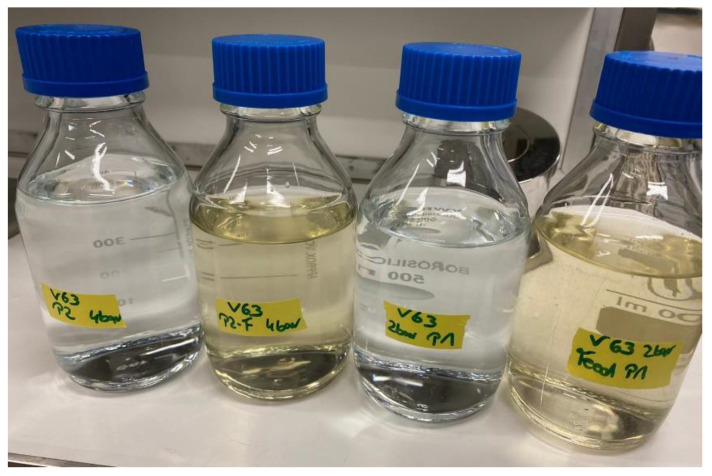
Photo of permeate and feed in an experiment with treated wastewater. From left to right: permeate at TMP = 4 bar, feed of the same experiment, permeate at TMP = 2 bar, and feed of the same experiment (Source: Oeltze).

In the experiments with treated wastewater, clear colour differences were detected between feeds and permeates and can be observed in Figure 7. The coloured molecules present in wastewater can have diverse sources, such as humic acids and ferric salts among others. During the filtration with hybrid ceramic membranes, coloured molecules present in treated wastewater were also retained.

### 3.3. Permeability and Flux

**Figure 8:**
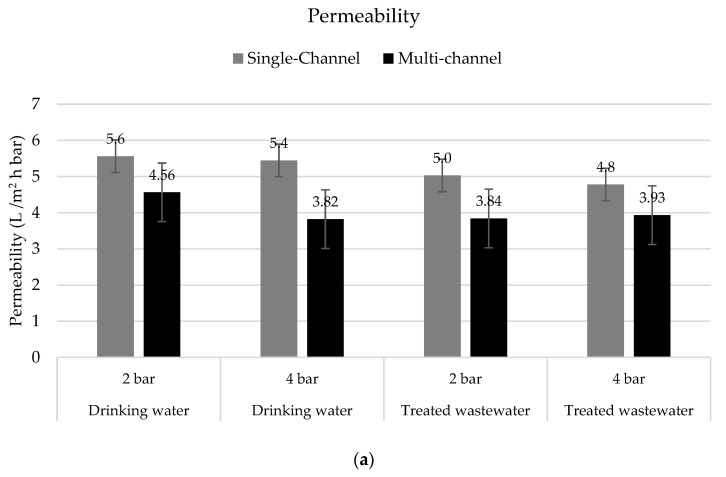

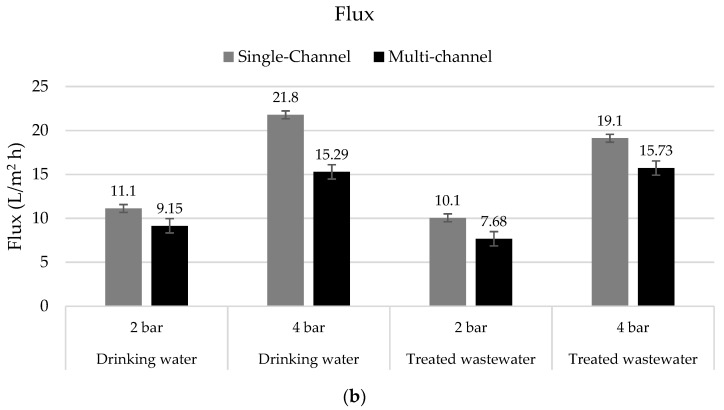
(**a**) Permeability in L/m^2^ h bar and (**b**) flux in L/m^2^ h in tests with drinking water (n = 3) and treated wastewater (n = 3–6) with single-channel hybrid membranes and multi-channel hybrid membranes under different TMPs at 15 °C.

Permeability is a key operational parameter used to determine the performance of membranes and the economic viabilities of their applications. The permeability values obtained with the single-channel membranes and the upscaling step with multi-channel hybrid membranes are compared in Figure 8.

It is clear that the permeability observed in the single-channel membranes was higher than the one observed in the multi-channel membranes. This was probably an effect of the membrane configurations as the resistances were higher in the multi-channel membranes. Due to the ceramic substrate designs, channel diameters, and resulting wall thicknesses between the utilized channels, the resistances for water to leave the ceramic segment increased. However, for a single-channel segment, the one channel was participating 100% in the filtration process and the individual channels for each row of a multi-channel segment contributed differently, leading to slightly different permeabilities compared to the single-channel design.

Moreover, the permeability obtained with the treated wastewater was on average lower than with the drinking water. The increased amount of solutes, the particulate matter, and their variety present in the treated wastewater appeared to influence the throughput of the membrane. However, the reductions in permeability were only 9% on average (max 16%).

Due to the membranes’ configurations, the flux was higher in the single-channel membranes, and as described before, the rejection of pharmaceuticals appeared to increase with the flux [31].

The permeabilities of the tested hybrid membranes were comparable to or slightly lower than those of membranes used for nanofiltration (NF) as informed by different authors [9,32,33,34] (between 1.4 and 7.8 L/m^2^ h bar) or even higher when compared with similarly coated hybrid membranes [35]. Even considering that the permeability was equal to or slightly lower than in NF applications, the presented technology has clear advantages, especially considering operational costs as the hybrid membranes can be operated at very low TMPs (i.e., 2 bar). In contrast, the NF membranes operate at TMPs of between 5 and 15 bar [36].

### 3.4. Cleaning

**Figure 9:**
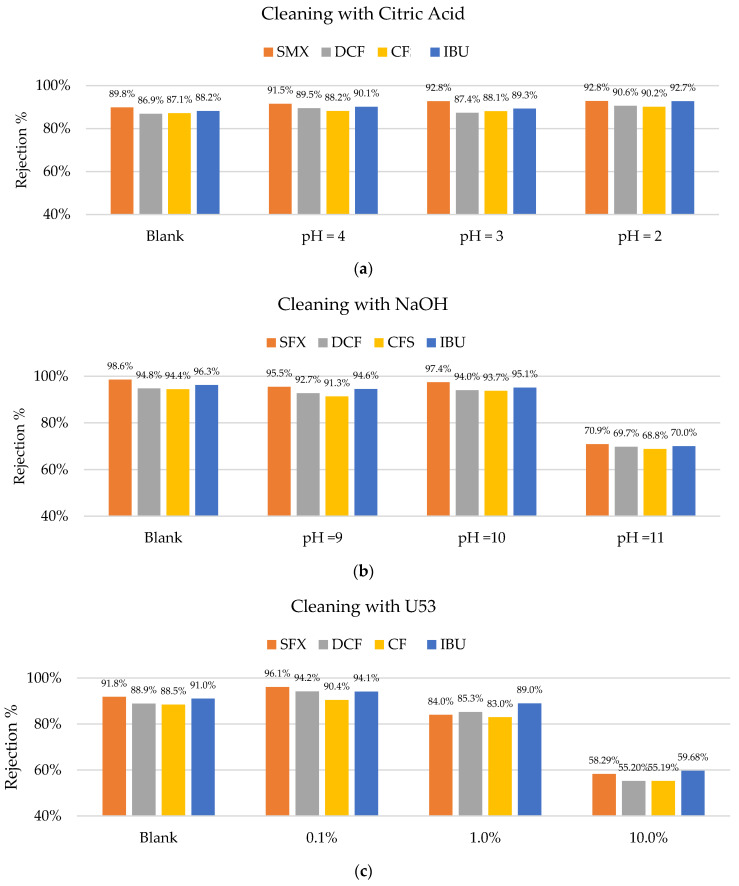

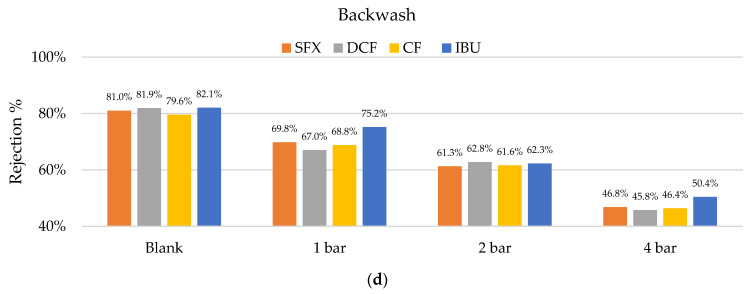
Rejection percentages of sulfamethoxazole (SMX), diclofenac (DCF), clofibric acid (CF), and ibuprofen (IBU): (**a**) Before cleaning (blank) and after cleaning with citric acid at pHs of 4, 3, and 2; (**b**) before (blank) and after cleaning with NaOH at pHs of 9, 10, and 11; (**c**) before and after cleaning with U53s of 0.1 to 10%; (**d**) and backwashes at different TMPs.

During the short-time operation of the membranes, no fouling was detected. Therefore, the cleaning procedures were carried out to test the LbL-coating durability and stability. Figure 9 shows that citric acid does not have a negative effect on the membrane performance up to a pH of 2. However, cleaning with NaOH showed a significant change in the membrane performance after cleaning at a pH of 11. Further research is required to understand which parts of the coating are affected by caustic solutions reaching pHs of 11. It is hypothesized that possibly PAH (polycation) was affected by the extremely caustic solution since its pKa is 8.5. At a pH as high as 11, the amine groups became deprotonated and lost their charges. This weakened the ionic bounds with the PSS (polyanion). However, the coating was not detached completely since there were also hydrogen bonds, which stabilized the coating. Due to the nature of the sulfone group of the PSS, there was no protonation at a low pH and no loss in charge. It was previously tested and determined that PDADMAC/PSS-coated membranes are resistant to cleaning with hypochlorite, which is not possible for any commercial nanofiltration membrane [19].

A similar effect was observed when using the enzymatic solution (U53). P3-Ultrasil 53 is an enzymatic cleaner containing mainly EDTA, SDS, and phosphates. The concentration of 10% affected the membrane’s performance negatively, significantly reducing the rejections of all the studied pharmaceuticals. The presence of SDS might have had a competitive effect on the PSS and therefore disturbed the polyelectrolytes coating, which is a possible explanation for the observed phenomenon, indicating that cleaning agents with ionic detergents are not suited for the cleaning of this type of membrane.

The backwash also had a negative effect on the filtration performance. Already at low a TMP (1 bar), the rejection was reduced from around 80% to below 70% on average. A TMP of 4 bar reduced the rejection of pharmaceuticals to below 50%. These results were also reflected in the permeability, which increased proportionally to the reductions in the rejection rates.

The backwash times were relatively long, with 10 min per pressure stage. Therefore, experiments with lower operational times could be performed to obtain a better understanding of the LbL-coating stability. Further experiments with membrane fouling should be performed in the future to determine the best combination of cleaning regimes for hybrid membranes and to test the limits of different LbL membrane coatings.

## 4. Conclusions

The LbL-coated ceramic membranes showed significant retentions of the tested pharmaceuticals. These results were confirmed in repeated experiments, both with high concentrations (with spiking) and low concentrations (without spiking). Moreover, high retentions of some common pollutants in treated wastewater (e.g., COD, TOC, and phosphorus) were observed.

The results imply that the application of hybrid ceramic membranes can contribute to an overall improvement in the quality of treated wastewater. This can help to meet increasingly strict discharge standards and also allow or improve the possibility of reusing treated wastewater. As the reuse of treated wastewater becomes increasingly important in different regions worldwide, this technology is highly promising to serve this purpose.

A common concern of the operation of membranes is their energy consumption mostly derived from the generation of large TMPs. The presented results were obtained at relatively low operating pressures (2 and 4 bar), which help to keep operating costs low in comparison with typical nanofiltration (TMP > 5 bar) and reverse osmosis (TMP > 10 bar) operations.

For the single-channel membranes, the retention for pharmaceuticals was considerably higher than for the multi-channel membranes. On the one hand, there was a lower flux measured for the multi-channel membranes, which might have influenced the rejection [31]. On the other hand, the single-channel membranes were studied much more during our experiments, and therefore, the coating procedure was much more optimized, which might have had an influence as well. It is relevant to mention that the efficiency of the membranes for the removal of pollutants was highly dependent on the operating conditions (fluxes, TMPs, and cross-flow velocities) and the conditions of the feed water (pHs, ion concentrations and compositions, and temperatures). These parameters should be carefully considered when evaluating the performance of hybrid ceramic membranes.

Moreover, no fouling or blockages of the membranes were detected during the test period. The stabilities and durabilities of the hybrid membranes against typical cleaning processes (acidic, caustic, and enzymatic solutions) and backwashes were tested. The hybrid membranes showed decreased performance after cleanings at pHs of >10. The enzymatic solution (U53) also challenged the performance of the hybrid membranes, even at low concentrations (0.1%). The main reason for this might have been the ionic detergent sodium dodecyl sulfate (SDS) in the enzymatic cleaning solution, which can compete with the anionic PSS in the coating. For hybrid membrane cleanings, SDS should be avoided. Backwash regimes for 10 min at 1 bar (flux = 13.3 L/m^2^ h) decreased the membranes’ performance. Further experiments are required to determine the ideal cleaning conditions for hybrid ceramic membranes. In large scale applications, a prior evaluation of the cleaning strategies will be necessary.

## Figures and Tables

**Table 1:** Characteristics of studied pharmaceuticals.

Pharmaceutical	MW	Net Charge	Topological Polar Surface	Solubility in Water	pKa	Source
	g/mol		Å^2^	mg/L		
Sulfamethoxazole	253.28	+	107	610 (37 °C)	1; 5.7	[21]
Diclofenac	296.15	−	49.3	2.37 (25 °C)	4.15	[22]
Clofibric acid	214.65	0	46.5	583 (20 °C)	3.18	[23,24]
Ibuprofen	206.28	−	37.3	21 (25 °C)	5.2	[25]

MW = Molecular weight.

**Table 2:** Filtration and cross-flow conditions of the rejection tests.

Type of Membrane	Filtration Area	Flux	Diameter	Area Channel	Crossflow Velocity
	m^2^	m^3^/s	m	m^2^	m/s
Single-channel	0.0075	1.11 × 10^−4^	0.0075	4.42 × 10^−5^	2.15
Multi-channel	0.35	1.11 × 10^−4^	0.0023	5.82 × 10^−4^	0.16

**Table 3:** Summary of operational parameters of the different experiments.

Membrane Type	Matrices	Additional Pharmaceuticals	TMP (bar)
Single-channel	Drinking water	Yes	2
	Treated wastewater	Yes	2
	Drinking water	Yes	4
	Treated wastewater	Yes	4
Multi-channel	Drinking water	Yes	2
	Treated wastewater	Yes	2
	Drinking water	Yes	4
	Treated wastewater	Yes	4
Multi-channel	Treated wastewater	No	2
	Treated wastewater	No	4

**Table 4:** Parameters for cleaning approaches.

Cleaning Type	pHs	Information
Citric acid	7	w/o
	4	-
	3	-
	2	-
NaOH	7	w/o
	9	-
	10	-
	11	-
Enzyme (P3-Ultrasil 53)	7.6	w/o
	7.5	0.1%
	7.7	1.0%
	7.8	10%
Backwash	7.6	w/o
	7.8	1 bar (10 min)
	7.7	2 bar (10 min)
	7.7	4 bar (10 min)

w/o = without chemical dosing or backwash.

**Table 5:** Measured concentrations in the experiments with and without spiking (n = 2).

Pharmaceutical Concentration	w/ Spiking				w/o Spiking		
	Feed (µg/L)	MU Feed	Permeate (µg/L)	MU Permeate	Feed (µg/L)	Permeate (µg/L)	MU
Sulfamethoxazole	87.923	11.9%	9.836	27.44%	5.005	0.758	36.8%
Diclofenac	108.390	4.9%	13.039	11.38%	10.580	2.185	32.0%
Clofibric acid	105.751	2.3%	14.623	9.42%	5.065	0.880	34.8%
Ibuprofen	91.383	3.8%	10.638	6.73%	3.750	0.658	16.3%

MU: Measurement uncertainty. MU represents the measurement error or deviation from standard values in HPLC (with spiking) or MS (without spiking) measurement methods.

## Data Availability

The data generated in the project can be requested from the corresponding author.

## Abbreviations

CIP	Cleaning in place
CF	Clofibric acid
COD	Chemical oxygen demand
DAD	Diode array detector
DCF	Diclofenac
HPLC	High-performance liquid chromatography
IBU	Ibuprofen
LbL	Layer–by-layer
LOD	Limit of detection
MS	Mass spectrometry
MW	Molecular weight
MWCO	Molecular weight cut-off
NF	Nanofiltration
PAH	Poly(allylamine hydrochloride)
PSS	Poly(styrenesulfonate)
SDS	Sodium dodecyl sulfate
SMX	Sulfamethoxazole
TMP	Transmembrane pressure
TOC	Total organic carbon
TP	Total phosphorous
U53	P3 Ultrasil 53, enzymatic membrane cleaner
WWTP	Wastewater treatment plants
